# Nose and Midface Augmentation by Rib Cartilage Grafts: Methods and Outcome in 32 Cases

**DOI:** 10.1155/2015/849802

**Published:** 2015-12-10

**Authors:** Adham Farouk, Saad Ibrahiem

**Affiliations:** Department of Plastic and Reconstructive Surgery, Faculty of Medicine, Alexandria University, Alexandria, Egypt

## Abstract

Recession of the midface is a relatively common condition that can have a negative impact on facial and nasal aesthetic appearance, and it poses a challenge to plastic surgeons. In cases with generalized maxillary retrusion and/or malocclusion, bone advancement surgery is required, but in localized cases, mostly seen in cleft lip patients, the quest is for an ideal material and a proper technique that would be used to augment the receding area. Throughout a period of seven years, thirty-two patients with nose and midface retrusion were managed by a construct of rib cartilage grafts designed to compensate the deficiency at the maxillary, piriform, and premaxillary areas. Results were satisfactory for most patients in terms of improved fullness of malar area, improved nasal tip projection and rotation, and improvement of upper lip. The presented technique of rib cartilage grafting is a safe and effective method for nose and midface augmentation.

## 1. Introduction

A wide variety of pathologic entities causes alteration of midfacial skeletal growth, resulting in varying degrees of midface deficiency presenting as maxillary retrusion with or without malocclusion. These include acquired conditions (posttraumatic and postsurgical) and congenital deformities; best known example (and most common as well) is the patients with cleft lip and palate, in whom the nature of the deformity (deficiency and hypoplasia of maxilla and piriform aperture) and the surgical procedures used to correct it take their toll on the final shape and projection of the midface. This condition is frequently accompanied by other deformities like nasal base recess, nasal tip ptosis, sunken cheeks, and deficient upper lip thrust [1–6].

Many surgical procedures have been suggested and various techniques have been described to address such deformities, with varying degrees of success and complications. These included surgical bone advancement, distraction osteogenesis, autologous osseous and cartilaginous grafts, homologous cartilaginous grafts, and alloplastic implants [1–4, 7–19].

This paper presents a technique of using rib cartilage grafts for nose and midface augmentation in a series of patients.

## 2. Patients and Methods

Thirty-two patients with maxillary hypoplasia, piriform deficiency, alar base recess, nasal tip ptosis, and deficient upper lip thrust, secondary to cleft lip anomaly, referred for correction of such deformities, throughout the period from 2007 to 2014, were subject to augmentation by rib cartilage grafts.

**Figure 1:**
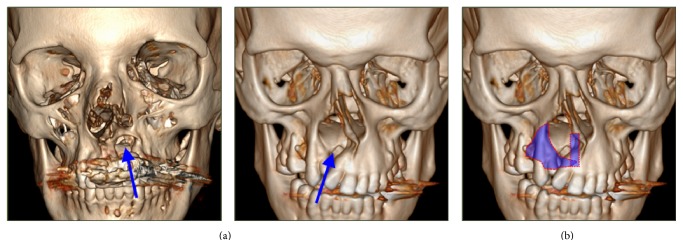
(a) Computed tomography pictures of two patients; arrows point to maxillary and piriform deficiency. (b) Computed tomography picture used to fashion the design of the cartilaginous construct (superimposed blue shapes).

Adequate preoperative analysis involved detailed history taking, thorough clinical examination, and three-dimensional computed tomography for accurate diagnosis of the skeletal defects (Figure 1(a)), and for adequate planning of the shape and position of the cartilaginous grafts required to augment those skeletal defects (Figure 1(b)).

Surgery was performed under general anesthesia, with local infiltration of the perimeter of the surgical fields (nasolabial and chest wall areas) with 1 : 10000 norepinephrine.

**Figure 2:**
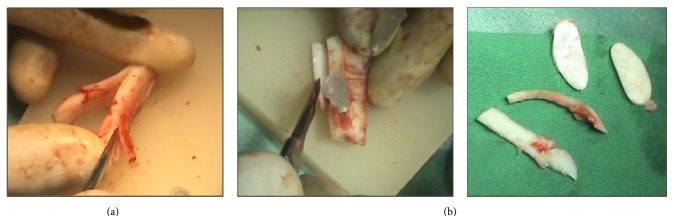
Carving rib cartilage grafts. (a) Carving a straight rod out of the central portion of rib cartilage specimen. (b) Carving thin plates out of the peripheral portion of rib cartilage specimen.

The cartilaginous part of the right sixth or seventh rib is harvested (by subperichondrial dissection) through a 5–7 cm transverse incision over the relevant area at the chest wall. Two types of grafts are then sculpted out of the harvested cartilage; the first is a straight (30–40 mm × 5 mm) rod graft carved from the central portion of the rib cartilage (Figure 2(a)), and the second is multiple 1–1.5 mm thickness plates with different shapes and surface areas carved from the remainder of the rib cartilage (Figure 2(b)).

**Figure 3:**
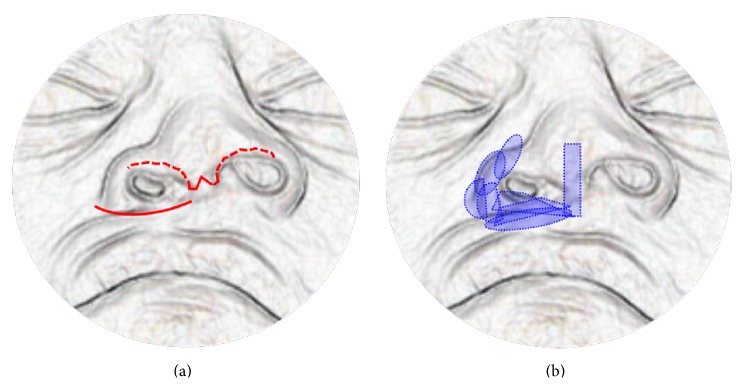
(a) Diagram illustrating the incision along the border of upper lip and nasal sill to expose the maxilla and piriform aperture, and the transcolumellar infracartilaginous incision to expose the nasal skeleton. (b) Diagram showing the planned shapes and arrangement of the cartilaginous implants.

The nasal skeleton was exposed through an external approach, and the piriform aperture and maxilla were exposed through an incision along the border of upper lip and nasal sill (Figure 3(a)). The above-mentioned cartilaginous grafts were then laid into a construct with two components: an anteromedial component comprising a suitable length of the straight rod graft and vertically oriented as a columellar strut and a posterolateral component comprising the plate grafts layered over each other in variable orientations according to the specific pattern needed for each particular case to augment the nasal floor, lateral nasal wall, and maxilla (Figure 3(b)).

**Figure 4:**
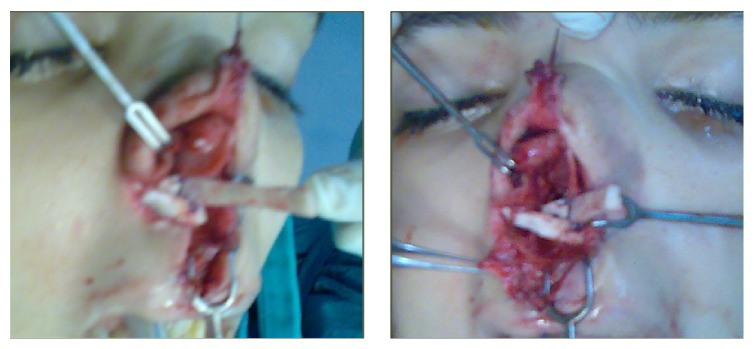
Two views during implantation of the rib cartilage construct.

The grafts are sutured to each other and fixed to medial crura of lower lateral cartilages, caudal end of septum, periosteum of anterior nasal spine, piriform aperture, and maxilla by permanent sutures (Figure 4). (Note that, in bilateral cases, the cartilaginous graft construct had 3 components: one anteromedial and two posterolateral components.)

Optional additional steps to remodel the nasal skeleton included hump resection, septoplasty, cephalic trims of alar cartilages, lobular alar cartilage incision and overlap, lateral crural steal or overlay, and interdomal tip refining sutures.

All patients received antibiotics for 10 days starting the night before the operation, and wash of surgical fields with gentamycin-saline solution was carried out throughout the surgery. Chest wall and nasal wounds were closed in layers, nasal cavities were packed for 3-4 days, and an external nasal splint was applied for 7–10 days.

## 3. Results

Out of the 32 patients enrolled for the described technique, 29 patients (90.6%) had unilateral augmentation and 3 patients (9.4%) had bilateral augmentation. They were 26 females (81.25%) and 6 males (18.75%). Their age ranged from 18 to 27 years and the mean age at the time of surgery was 21.9 years. The number of previous surgeries (lip/palate/nose) ranged from 1 to 6 surgeries (mean: 2 surgeries).

Throughout the follow-up period that ranged from 6 months to 7 years (mean: 47.5 months), surgical results were satisfactory to most patients and only one case required a revision rhinoplasty for refinement of nasal dorsum after 2 years.

**Figure 5:**
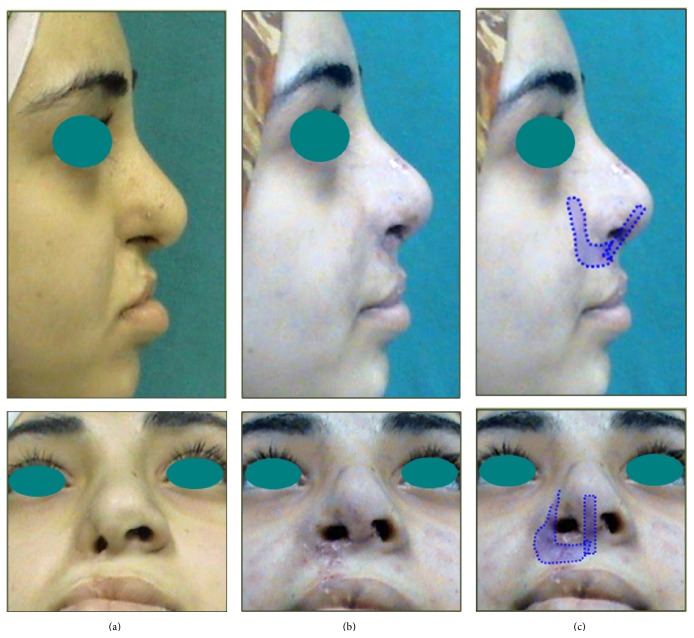
Example (1) for clinical results of rib cartilage grafts. (a) Preoperative views of a patient with recession of nasal floor, alar base, and malar area, and obtuse nasolabial angle. (b) Two-week postoperative views showing improved receding elements, nasal tip rotation, and upper lip thrust. (c) Superimposed blue shapes demonstrate the relative size and position of the cartilaginous implants.

**Figure 6 fig6:**
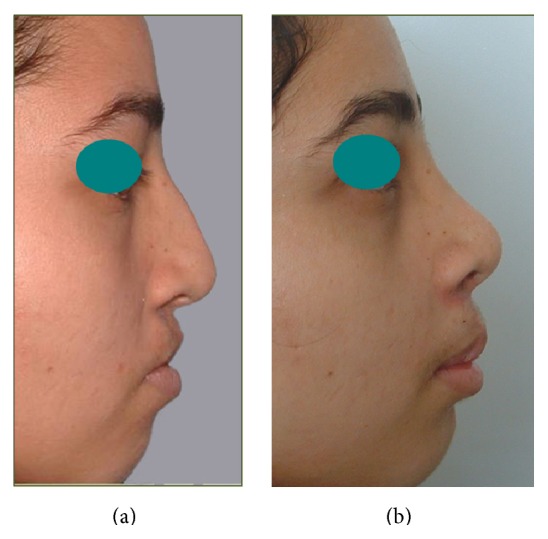
Example (2) for clinical results of rib cartilage grafts. (a) Preoperative views of a patient with premaxillary and maxillary retrusion. (b) Three-year postoperative views showing augmented midface, nasal rotation, and upper lip thrust.

**Figure 7:**
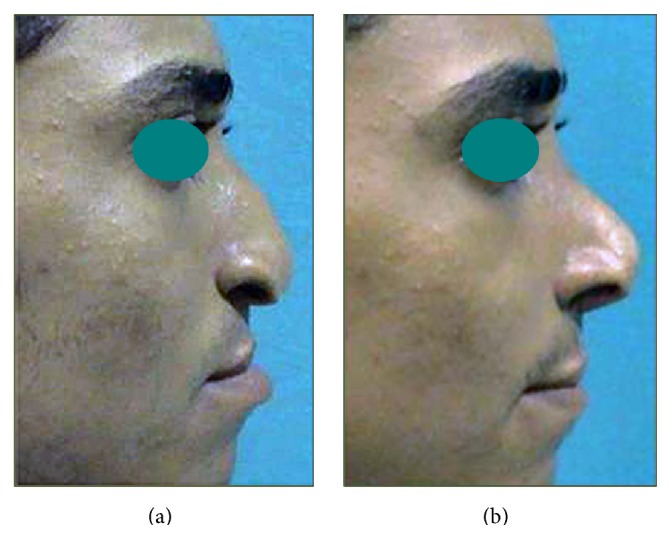
Example (3) for clinical results of rib cartilage grafts. (a) Preoperative views of a patient with premaxillary depression and droopy nasal tip. (b) Two-year postoperative views showing augmented midface, nasal rotation, and upper lip thrust.

Figures 5 [Fig fig6]–7 depict 3 examples to the outcome of rib cartilage grafts for nose and midface augmentation. These pictures illustrate significant improvement of midface deficiency, nasal tip projection/rotation, and upper lip thrust, and the basal view of Figure 5 demonstrates distinctive improvement of alar base position and nasal sill morphology.  Figure 5(c) illustrates the relative size and position of the cartilaginous implants.

**Figure 8:**
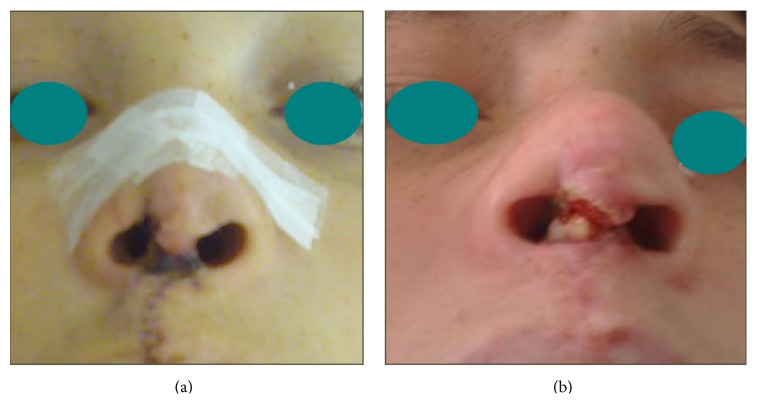
Picture of a complication which occurred. (a) Skin necrosis at distal part of the columellar flap. (b) Exposure of a part of the rib cartilage graft.

Minor complications occurred in 2 patients (6.25%) in the form of hypertrophic scarring of the chest wall wound, which responded well to topical therapy without any need for surgical scar revision, and in 1 patient (3.1%), necrosis at the columellar wound edge occurred and resulted in exposure of a part of the cartilage graft (Figure 8) but did not mandate its extraction and eventually healed by secondary intention.

## 4. Discussion

Recession of the midface is a relatively common condition that can have a negative impact on facial and nasal aesthetic appearance, and it poses a challenge to plastic surgeons. In the less frequent cases of midface deficiency that exhibits generalized retrusion of the maxilla (retrognathic maxilla) and/or malocclusion, distraction osteogenesis, orthognathic surgery, or intraoral maxillary expansion is indicated, but in the more frequent cases exhibiting localized retrusion without malocclusion, characteristically seen in, but not limited to, patients with cleft lip deformity, the quest is for an ideal material and a proper technique that would be used to augment the receding area.

Regarding the materials (implants) that can be used for nose and midface augmentation, they fall into three categories according to their source: autologous implants (costal cartilage or calvarial bone), homologous implants (irradiated costal cartilage, acellular dermis), and alloplastic implants (silicone, polyethylene, polytetrafluoroethylene, polyesters, and polyamides). The main advantages of homologous cartilage and alloplastic implants are the unlimited supply and the absence of donor site morbidity, yet the significant disadvantages of possible autoimmune reactions, extrusion, infection, high cost, and patients' concerns about their artificial nature and their safety diminish the odds of their appropriateness for use in this purpose [11, 20–24]. Therefore, the biocompatibility of autologous implants is the main overweighting factor for their selection in this respect.

In comparison to costal cartilage grafts, bone grafts are more difficult to harvest, shape, and fix, are more prone to resorption, and have more potential significant donor site morbidity [23, 24]. This being said, we believe that rib cartilage graft is the most feasible choice for use in the purpose of midface augmentation. It has the lowest rates of rejection, resorption, and infection, is easily harvested and sculpted, and is available in plentiful supply, and our surgical technique adopted specific tactics and precautions that would optimize the results and minimize its drawbacks and side effects.

Strictly aseptic surgical conditions under a strong antibiotic umbrella and liberal wash of surgical fields with gentamycin-saline solution and starting the operation with the process of cartilage graft harvesting, shaping, and soaking in antibiotic-saline solution minimized potential contamination from nasal mucosa and kept infection rate nil in our cases.

Delaying closure of the chest wall wound to allow returning of any remaining pieces of cartilage into the costal defect, and closure of the perichondrium followed by meticulous closure of the wound in layers, prevented the incidence of any significant donor site morbidity.

To maximize survival of the implanted cartilage, and also to make it more amenable to fit onto the irregular defects of the premaxilla, piriform margins, and maxilla, we used thin (1–1.5 mm) slices and stacked them in layers sutured to the periosteum and to each other. Though the most significant drawback of rib cartilage grafts, which is cartilage “warping,” is not a big concern in “rough volume augmentation” of midface defect as it would be in “fine shaping” of nasal dorsum, still, the thin slicing can neutralize the mechanical vectors and minimize the impact of any possible warping, and for the columellar strut that is used to correct the droopy nasal tip and nasolabial angle, we employed the most commonly applied method for combating cartilage warping by obtaining balanced cross section from the center of the rib cartilage specimen.

Most of the patients were satisfied by the results and only one of the earlier cases in this series developed a significant complication in the form of exposure of part of the cartilage graft that healed with secondary intention and this complication was attributable to a misfortunate wound edge necrosis (at the most distal part of the columellar flap) and not attributable to an inherent defect in the technique.

## 5. Conclusions

The described technique of rib cartilage grafting is a safe, versatile tool for nose and midface augmentation. It can be a good and relatively simple alternative to orthognathic and distraction surgeries in cases of cleft lip deformity without malocclusion, and in cases with malocclusion it can be a complementary procedure to those surgeries when their results are insufficient. The described technique yields not only augmentation of the alar base and malar area retrusion but also improvement of nasal tip projection/rotation and upper lip thrust; that is, it optimizes the aesthetic appearance of all the components of the midface region.
